# Granisetron versus Granisetron-Dexamethasone for Prevention of Postoperative Nausea and Vomiting in Pediatric Strabismus Surgery: A Randomized Double-Blind Trial

**DOI:** 10.1155/2016/4281719

**Published:** 2016-01-26

**Authors:** Renu Sinha, Dilip Shende, Souvik Maitra, Neeraj Kumar, Bikash Ranjan Ray, Virender Kumar Mohan

**Affiliations:** ^1^Room Number 376, Dr. R. P. Centre for Ophthalmic Sciences, AIIMS, New Delhi, India; ^2^Room Number 374, Dr. R. P. Centre for Ophthalmic Sciences, AIIMS, New Delhi, India; ^3^Room Number 5011, Department of Anaesthesiology, AIIMS, New Delhi, India; ^4^Room Number 5007, Department of Anaesthesiology, AIIMS, New Delhi, India

## Abstract

*Aim.* Efficacy of granisetron and combination of granisetron and dexamethasone was evaluated for prevention of postoperative nausea and vomiting (PONV) in children undergoing elective strabismus surgery.* Methods.* A total of 136 children (1–15 years) were included. Children received either granisetron (40 mcg/kg) [group G] or combination of granisetron (40 mcg/kg) and dexamethasone (150 mcg/kg) [group GD]. Intraoperative fentanyl requirement and incidence and severity of oculocardiac reflex were assessed. PONV severity was assessed for first 24 hours and if score was >2, it was treated with metoclopramide. Postoperative analgesia was administered with intravenous fentanyl and ibuprofen.* Results.* The demographic profile, muscles operated, and fentanyl requirement were comparable. Complete response to PONV in first 24 hours was observed in 75% (51/68) of children in group G and 76.9% (50/65) of children in group GD, which was comparable statistically (*p* = 0.96, Fisher exact test; OR 1.11, 95% CI 0.50, 2.46). Incidence of PONV between 0 and 24 hours was comparable. One child in group G required rescue antiemetic in first 24 hours and none of the children had severe PONV in group GD. There was no significant difference in incidence or severity of oculocardiac reflex.* Conclusion.* Dexamethasone did not increase efficacy of granisetron for prevention of PONV in elective pediatric strabismus surgery. Registration number of clinical trial was CTRI/2009/091/001000.

## 1. Introduction

Postoperative nausea and vomiting (PONV) are a frequent and important cause of morbidity following pediatric strabismus surgery [1]. The incidence of PONV without any prophylaxis ranges from 41% to 88% [2, 3]. PONV after strabismus surgery are the main cause for readmission which increase the total medical expenses due to need for both rescue medication and prolonged hospital stay [1]. PONV may also lead to fear of visit to the hospital during subsequent surgeries.

Granisetron, a selective 5-hydroxytryptamine type 3 (5-HT_3_) receptor antagonist, is a potent and long acting antiemetic [4]. Review of literature showed that optimal dose of granisetron for prevention of PONV was 40 mcg/kg [5] while dexamethasone has been used in 50 mcg/kg to 1 mg/kg for PONV prophylaxis [6, 7].

It has been observed that combination of granisetron 40 mcg/kg and dexamethasone 150 mcg/kg is more effective than granisetron 40 mcg/kg alone in children undergoing various types of surgeries [5, 8]. However, there is no clinical trial to evaluate the efficacy of this combination in pediatric strabismus surgery alone. Hence, this prospective, randomized, double-blind clinical trial was designed to evaluate the efficacy of granisetron as compared to combination of granisetron and dexamethasone in children undergoing elective strabismus surgery.

## 2. Methods

After institutional ethical committee approval and parental consent, 136 children with American Society of Anesthesiologists (ASA) Physical Statuses I and II, aged 1 to 15 years, scheduled for elective strabismus surgery under general anaesthesia were included in this prospective, randomized, double-blind clinical trial. This trial has been registered under Clinical Trail Registry-India (CTRI/2009/091/001000 [registered on 11/12/2009]). This study was conducted from 16/09/2009 to 20/12/2010.

Children with an obviously difficult airway and mental retardation and taking drugs that have antiemetic action (phenothiazines, corticosteroids, and tricyclic antidepressant) and with previous history of motion sickness were excluded from the trial. Parents were informed about nausea, retching, and vomiting criteria. Nausea was not assessed in children less than 6 years of age. Retching was defined as laboured, spasmodic rhythmic contraction of respiratory muscle without any expulsion of gastric contents and vomiting was defined as the forceful expulsion of gastric contents from the mouth.

Children were allowed for oral solid or liquid intake till 6–8 hours and for clear fluid till 2 hours before surgery. None of the children received premedication. In the operating room, standard monitoring [heart rate (HR), electrocardiography (ECG), oxygen saturation (SpO_2_), and noninvasive blood pressure (NIBP)] was applied. Anaesthesia was induced with oxygen (O_2_) and nitrous oxide (N_2_O) (1 : 1) and incremental concentration of sevoflurane. Following intravenous cannulation, fentanyl 2 mcg/kg as well as vecuronium 0.05 mg·kg^−1^ was administered to facilitate insertion of appropriate size flexible laryngeal mask airway (LMA). Controlled ventilation with O_2_ and N_2_O (1 : 1) isoflurane to maintain minimal alveolar concentration between 1.0 and 1.3 was initiated through close circuit to maintain end tidal carbon dioxide (EtCO_2_) between 35 and 45 mm Hg. Children were randomly allocated to one of the two groups using computer-generated randomization list and opaque sealed envelopes. Envelope was opened by the anesthesiologist who was not involved in the study and prepared the study drugs to a fixed volume of 5 mL for PONV prophylaxis. The anesthesiologist and observer were not aware of the group of the children. Children in group G received granisetron 40 mcg/kg and group GD received granisetron 40 mcg/kg plus dexamethasone 150 mcg/kg intravenously before start of surgery. Topical proparacaine 0.5% was instilled in the operating eye before incision. For postoperative analgesia, paracetamol (PCM) suppository in the dose of 30–40 mg/kg was administered before the start of surgery.

After incision, the frequency and severity of oculocardiac reflex (OCR) were recorded and treated accordingly. For any increase in HR and/or mean arterial pressure (MAP) by >20%, rescue analgesia with IV fentanyl 0.5 mcg/kg was administered. Intraoperative fluid was administrated according to standard protocol. Any other intraoperative complication was noted and treated. At the end of surgery, after return of respiration neostigmine 0.05 mg/kg as well as glycopyrrolate 0.01 mg/kg was administered to reverse the neuromuscular blockade and LMA removed. The child was put in left lateral position and transferred to the postanaesthesia care unit (PACU) when he was able to maintain airway. Recovery was evaluated by Modified Aldrete's postanaesthesia recovery score and time required to achieve a score of 10 was noted [9].

PONV and pain were assessed by PONV score [10] and modified objective pain score (MOPS) system [11], respectively, in PACU and ward by a nurse (who was unaware about the antiemetic used) continuously and recorded at arrival in PACU, at spontaneous awakening, and at 1 hr, 2 hrs, shifting to ward, 6 hrs, and 24 hrs. The children were kept in step 1 PACU once awake and shifted to step 2 PACU for minimum of 2 hours after anaesthesia and shifted to the ward once they were pain-free with stable vital signs and no nausea or vomiting for at least 1 hour whichever was earlier. PONV were scored “0” no nausea, retching, or vomiting, “1” nausea or retching, “2” one vomiting episode in 30 min, and “3” persistent nausea (>30 min) or two or more vomiting episodes in 30 min. Incidence and severity of PONV (see Table 3) were noted and if PONV score was >2, IV metoclopramide 150 mcg/kg was administered. Total number of vomiting episodes as well as complete response (no nausea or vomiting in 24 hours) was also noted.

Pain was assessed continuously by MOPS. If score was >3, rescue fentanyl 0.5 mcg/kg IV was administered and recorded. In the ward, when child complained about pain or MOPS was >3, syrup ibuprofen 10 mg/kg was given. Parents were asked to rate overall satisfaction at 0-to-10-point scale at the end of 24 hours.

The number of patients required for the study was calculated on assumption that addition of dexamethasone to granisetron will result in decrease in the incidence of PONV from 20% to 3.3% [5]. For *α* = 0.05 and power (1 − *β*) = 0.8, based on these assumptions, 59 children in each group were required. We enrolled 68 children in each group with an anticipation of 15% dropout.

Statistical analysis was carried out using Stata 9.0 (College Station, Texas, USA). Data were presented as number (percentage), mean ± SD, or median as appropriate.

Sex ratio, ASA physical status, number of children requiring postoperative fentanyl and ibuprofen, and incidence of PONV were analyzed by Pearson chi-square (Fisher's exact) test; odds ratios (OR) with 95% confidence interval were also calculated.

Demographic data including age, weight, duration of surgery and anaesthesia, number of muscles operated, intraoperative fentanyl requirement, Aldrete score at the time of shifting to PACU or time taken to reach the score of 10, and parents satisfaction score were analyzed by Wilcoxon rank sum test (Mann-Whitney *U* test). A two-tailed *p* value < 0.05 was considered statistically significant.

## 3. Result

Data was incomplete in 3 children in group GD; that is, final analyses were done in 68 children in group G and 65 in group GD.

Demographic data including age, sex, weight, and ASA physical status were comparable in both groups (Table 1). Number of muscles operated, intraoperative requirement of fentanyl, and duration of surgery and anaesthesia were also comparable in both groups (Table 1). There was no significant difference in the Aldrete score at the time of shifting to PACU or time taken to reach the score of 10 in both groups (Table 2). Postoperative requirements of fentanyl in PACU and ibuprofen in the ward were comparable in both groups and parent satisfaction score was also comparable (Table 2).

Complete response to PONV in 24 hours was observed in 75% (51/68) of children in group G and 76.9% (50/65) of children in group GD, which was comparable statistically (*p* = 0.96, Fisher exact test; OR 1.11, 95% CI 0.50, 2.46). In first 2 hours after surgery, 11.7% (8/68) of children experienced PONV in group G in comparison to 18.5% (12/65) of children in group GD and this is also statistically similar (*p* = 0.40, OR 1.70, 95% CI 0.65, 4.47). In group G, higher number of children 19.1% (13/68) experienced PONV in 2–6 hours in comparison to 10.8% (7/65) of children in group GD; however statistical significance has not been reached (*p* = 0.27, OR 0.51, 95% CI 0.19, 1.37). Analyzing the severity of PONV we found that, in 2–6 hours, group G, 14.71% (10/68) of children experienced nausea or retching in comparison to 6.15% (4/65) of children in group GD (*p* = 0.1) and vomiting is experienced by 3 children each in group G and group GD (*p* = 0.9). At 6–24 hours, nausea was experienced by 4.41% (3/68) of children in group G while there was none in group GD (*p* = 0.2); vomiting occurred in one-one child in both groups (*p* > 0.9). One child in group G required rescue antiemetic in PACU. Incidence of PONV was similar in all age groups in both groups (Table 4). Complications in both groups were minimal.

There was a strong correlation between number of muscles operated and OCR incidence but OCR incidence had no relation with occurrence of PONV.

## 4. Discussion

In the present study, we did not find any statistical increase in the efficacy of granisetron in prevention of PONV with dexamethasone in pediatric strabismus surgery [complete response to PONV in group G (75%) versus group GD (76.92%)]. These results are contradictory to the previous studies which showed 12–30% increase in efficacy of granisetron with dexamethasone combination [5, 8].

In the present study, complete response to PONV with granisetron was also decreased in comparison to previous studies [75% versus 80–84%] [5, 8]. The difference in results from previous studies could be attributed to higher incidence of PONV in strabismus surgery due to oculoemetic reflex and postoperative distortion of vision [12, 13]. Previous studies showing increased efficacy of granisetron with combination of dexamethasone in children were done in middle ear surgery and tonsillectomy [5, 8]. Fujii et al. in several trials found that granisetron and dexamethasone combination is superior to granisetron alone for PONV prophylaxis in patients undergoing breast surgery, thyroidectomy, and laparoscopic cholecystectomy. Subsequently, all the clinical trials by Fujii et al. were retracted. We too felt that well designed trails are required to know whether there is any clinical benefit of adding dexamethasone along with granisetron or not.

Variable results with the same drugs combination in different studies could be due to difference in study design, patient population, types of surgery, surgical techniques, duration of surgery, and anaesthesia techniques. Anaesthesia related factors include fasting time, premedication, induction techniques, use of N_2_O or high concentration of O_2_, endotracheal tube or supraglottic device, spontaneous or controlled ventilation, halothane or sevoflurane, and use of opioid or regional blocks [14]. Different doses of drugs are one of the major reasons for variable results, as Riad and Marouf [15] have used granisetron at a dose of 10 mcg/kg and dexamethasone at 500 mcg/kg after pediatric strabismus surgery which is different from the doses used in the present study (granisetron at 40 mcg/kg and dexamethasone at 150 mcg/kg). Gupta and Jain used granisetron at a dose of 40 mcg/kg for PONV prophylaxis after breast cancer surgery [16]. Dexamethasone has also been used at different doses (50 mcg/kg–1 mg/kg) for PONV prophylaxis and variable results were found [6, 7]. de Orange et al. [17] used dexamethasone at a dose of 150 mcg/kg in children, and we also used same dose in our study. Dose response relationship is another determinant of clinical effect of any drug; however, dose response relationship is questionable for granisetron both for PONV prophylaxis and for chemotherapy induced vomiting [18, 19]. Similar findings have been reported with dexamethasone also; De Oliveira Jr. et al. [20] in a meta-analysis found that 4 mg to 5 mg dose of dexamethasone seems to have similar clinical effects on the reduction of PONV as the 8 mg to 10 mg dose when dexamethasone was used as a single drug or as a combination therapy.

In the present study, we tried to avoid the confounding factors including opioid administration. As different opioids also have different emetogenic potential, in the present study, statistically similar doses of fentanyl were used in both groups. On the contrary, Riad and Marouf [15] did not mention whether any additional intraoperative opioid other than at the time of induction was used or not. Gombar et al. [5] used postoperative morphine; however they did not mention total morphine requirement in each group.

In different studies, age of the patients has been variable from 1 year to 40 years [8, 10]. We included children between 1 and 15 years and after statistical analysis we found that the incidence of PONV in different age groups was similar; however documentation of nausea may be less due to presence of 1/3 children below 6 years.

In the present study, we did not find any correlation of PONV with the incidence of OCR and the number of muscles operated which was similar to the previous study [21].

In our study, addition of dexamethasone reduced the number of episodes of vomiting in group GD (4/15) as compared to group G (8/17). Henzi et al. also found that dexamethasone and granisetron combination decreased late nausea and vomiting as compared to granisetron alone [22].

In a meta-analysis by Shen et al. dexamethasone and ondansetron have been found to be more effective at reduction of PONV in comparison to ondansetron after strabismus surgery [23]. As granisetron is more efficacious than ondansetron in prevention of PONV [24, 25], it is possible that dexamethasone cannot further enhance the efficacy of granisetron in strabismus surgery as seen in our study. Again, efficacy of any drug in preventing an undesirable response also depends upon the baseline risks of the patients. We have discussed the possible anaesthesia related risk factors in these patients and also we have tried to minimize them in our trial design. So, inherent risk of PONV in our patients is less than that of previous studies. As the events become rarer, a larger sample size will be required to obtain a statistical significance. Determination of baseline risk in these populations is difficult because designing a trial keeping a placebo group is not ethical in view of high incidence of PONV after strabismus surgery. Carlisle [26] in 2012 conducted a meta-analysis to know the impact of trials by Fujii et al. and found that after removing such trials there is no evidence of synergism between any antiemetics. Moreover exact mechanism of dexamethasone for PONV prophylaxis is not known; Chatterjee et al. [27] mentioned, “likely mechanisms are prostaglandin inhibition in peripherally, with facilitation of serotonergic antagonism and endorphin release centrally.”

Limitation of our study was that we have not studied the effects of this combination on late onset PONV as in our institute the children were discharged next day after the surgery.

Perioperative single use of dexamethasone may cause number of side effects including perioperative hyperglycemia, infection, delayed wound healing, tumor lysis syndrome, gastric ulceration, musculoskeletal problems, and neuropsychiatric manifestations [28].

## 5. Conclusion

The present study showed that efficacy of granisetron in prevention of PONV was not increased when it is combined with dexamethasone in elective pediatric strabismus surgery.

## Figures and Tables

**Table 1:** Patient's demographic profile.

	Group G (*n* = 68)	Group GD (*n* = 65)	*p* value
Age, years	7.4 (3.8)	7.4 (3.8)	0.97^£^
1–5	22	23	
5–10	29	26	
10–15	17	16	
Sex			
M : F	32 : 36	40 : 25	0.12^¥^
Weight, kg	20 [5–70]	20 [9–50]	0.91^€^
ASA status I/II	65/3	59/6	0.32^¥^
Number of muscles, median	2 [1–4]	2 [1–4]	0.09^€^
Duration of anaesthesia (min)	53.8 (14.9)	55.7 (16.6)	0.49^£^
Duration of surgery (min)	39.9 (13.9)	41.8 (15.7)	0.45^£^

ASA: American Society of Anesthesiologists.

Group G: children received granisetron at a dose of 40 mcg/kg.

Group GD: Children received granisetron at 40 mcg/kg and dexamethasone at 150 mcg/kg.

*Values are mean (SD), median [range], or number.*

^£^Independent sample *t*-test.

^¥^Chi-square test.

^€^Mann-Whitney *U* test.

**Table 2:** Analgesic requirement, Aldrete score, and parent satisfaction score. Values are in mean ± SD, number (percentage), or median [range].

	Group G (*n* = 68)	Group GD (*n* = 65)	*p* value
Intraoperative fentanyl, mcg	40 [10–150]	40 [15–130]	0.79^€^
Aldrete score at the time of shifting	8 [4–10]	9 [6–10]	0.34^€^
Time to reach Aldrete score of 10 (min)	15 [0–70]	15 [0–70]	0.19^€^
Children requiring fentanyl in PACU	8 (11.7%)	11 (16.9%)	0.39^¥^
Children requiring postoperative ibuprofen	46 (82.3%)	37 (56.9%)	0.20^¥^
Shifting time from PACU (min)	72.5 [30–180]	60 [20–180]	0.08^€^
Parent satisfaction score	8.3 ± 0.9	8.6 ± 0.9	0.07^£^

Values are mean (SD), median [range], or number.

Group G: children received granisetron at a dose of 40 mcg/kg.

Group GD: children received granisetron at 40 mcg/kg and dexamethasone at 150 mcg/kg.

PACU: postanaesthesia care unit.

SD: standard deviation.

^£^Independent sample *t*-test.

^¥^Chi-square test.

^€^Mann-Whitney *U* test.

**Table 3:** Incidence and severity of postoperative nausea vomiting (PONV) in different time interval (0–24 hours).

PONV score/number of children	Group	0	1	2	3	*p* value
0–2 hrs	Group G (*n* = 68)	60 (88.24%)	2 (2.94%)	5 (7.35%)	1 (1.47%)	0.3^*∗*^
	Group GD (*n* = 65)	53 (81.54%)	6 (9.23%)	6 (9.23%)	0	

2–6 hrs	Group G (*n* = 68)	55 (80.88%)	10 (14.71%)	3 (4.41%)	0	0.3^*∗*^
	Group GD (*n* = 65)	58 (89.23%)	4 (6.15%)	3 (4.62%)	0	

6–24 hrs	Group G (*n* = 68)	64 (94.12%)	3 (4.41%)	1 (1.47%)	0	0.2^*∗*^
	Group GD (*n* = 65)	64 (98.46%)	0	1 (1.54%)	0	

0–24 hrs^€^	Group G (*n* = 68)	51^¥^ (75%)	15^£^ (22.06%)	9^¢^ (13.24%)	1^*µ*^ (1.47%)	0.6^*∗*^
	Group GD (*n* = 65)	50^¥^ (76.92%)	10^£^ (15.56%)	10^¢^ (15.56%)	0^*µ*^	

Group G: children received granisetron at a dose of 40 mcg/kg.

Group GD: children received granisetron at 40 mcg/kg and dexamethasone at 150 mcg/kg.

^*∗*^Chi-square test.

€ does not represent total number of children.

¥ represents number of children who had complete response (no PONV between 0 and 24 hrs).

£ represents number of children who had nausea at some point of time between 0 and 24 hrs.

¢  represents number of children who had vomiting at some point of time between 0 and 24 hrs.

*µ* represents number of children who needed antiemetic at some point of time between 0 and 24 hrs.

**Table 4:** Incidence of postoperative vomiting (1–5 yrs) and postoperative nausea and vomiting (PONV) (5–15 yrs).

Age groups/number of children	Group G *n* = 68 (%)	Group GD *n* = 65 (%)	*p* value
1–5 years	3/22	4/23	>0.9^€^
5–10 years	8/29	9/26	0.6^¥^
10–15 years	6/17	2/16	0.3^€^

Group G: children received granisetron at a dose of 40 mcg/kg.

Group GD: children received granisetron at 40 mcg/kg and dexamethasone at 150 mcg/kg.

^¥^Chi-square test.

^€^Fisher exact test.
